# Protective effect of Urtica dioica L against nicotine-induced damage on sperm parameters, testosterone and testis tissue in mice

**Published:** 2014-06

**Authors:** Cyrus Jalili, Mohammad Reza Salahshoor, Ali Naseri

**Affiliations:** 1 *Fertility and Infertility Research Center, Kermanshah University of Medical Sciences, Kermanshah, Iran.*; 2 *Kermanshah University of Medical Sciences, Kermanshah, Iran.*

**Keywords:** *U.dioica L*, *Nicotine*, *Sperm parameters*, *Mice*

## Abstract

**Background:** Nicotine consumption can decrease fertility drive in males by inducing oxidative stress and DNA damage. Urtica dioica L (U.dioica) is a multipurpose herb in traditional medicine for which some anti-oxidative and anti-inflammatory properties have been identified.

**Objective:** The main goal is to investigate whether the U.dioica could inhibit nicotine adverse effects on sperm cells viability, count, motility, and testis histology and testosterone hormone.

**Materials and Methods: **In this study, hydro-alcoholic extract of U.dioica was prepared and various doses of U.dioica (0, 10, 20, and 50 mg/kg) and U.dioica plus nicotine (0, 10, 20, and 50 mg/kg) were administered intraperitoneally to 56 male mice for 28 consequent days. These mice were randomly assigned to 8 groups (n=7) and sperm parameters (sperm cells viability, count, motility, and morphology), testis and prostate weight, testis histology and testosterone hormone were analyzed and compared.

**Results: **The results indicated that nicotine administration (0.5 mg/kg) significantly decreased testosterone level, count and motility of sperm cells, and testis weight compared to control group (p=0.00). However, increasing the dose of U.dioica significantly boosted motility, count, normal morphology of sperm cells, seminiferous tubules diameter, and testosterone in all groups compared to control (p=0.00) and testis weight in 20 and 50 mg/kg doses in comparison with control group (p=0.00).

**Conclusion:** It seems that U.dioica hydro-alcoholic extract administration could increase the quality of spermatozoa and inhibits nicotine-induced adverse effects on sperm parameters.

## Introduction

Infertility is a health issue that has adverse effects in personal, social, and economic domains and is observed in 10-15% of the couples (1). About 40% of infertility problems are associated with men (2). Infertility in males is mostly due to sperm cells dysfunctions such as low sperm cells count, immaturity, abnormality, and lack of motility (3). 

Various studies have shown that consumption of nicotine-containing compounds decrease the sperm cells count and motility (4). Nicotine is a highly toxic organic compound containing nitrogen and alkaloid which is mostly found in tobacco (5). Nicotine can easily pass through the cell membrane and react to tubulin protein present in the cytoplasm of multiplying cells and cause cell division disorder (6). Some of studies have indicated that nicotine can damage sperm cell membrane and DNA and induce apoptosis in interstitial cells in testis (7).

Urtica dioica (U.dioica), in folk medicine, is used as an agent causing excessive secretion of urine, setting the operating cycle, as well as an astringent, an adrenal tonic, and gland balancer and anti-inflammatory agent (8). Gülçin *et al* showed that U.dioica infusion exhibits antioxidant capacity against phospholipids oxidation and acid linoleic resulting from iron (9). U.dioica is a plant containing essential amino acids, vitamins, and numerous nutrients (10). İşler *et al *has reported the anti-oxidant properties, anti-mutation and anti-tumor effects for U.dioica (11). One of the most important compounds and anti-oxidant of U.dioica root is flavonoides. This family of compounds have anti-oxidant activities and can modify some specific enzymes, so they can inactive some agents such as nitrite peroxide and hydroxide radical (12). 

Therefore, the present study was conducted to analyze protective effect of U.dioica on damage induced by nicotine in sperm parameters such as motility, count, viability, morphology, and also testis tissue, seminiferous tubules diameter, and testosterone in male mice.

## Materials and methods

**U.dioica** **L** **and Nicotine preparation**

In this experimental study, U.dioica is a plant belonging to Urticaceae plant family. Its seeds are widely used in folk medicine in many parts of Iran. This plant was purchased from a traditional medicine center and identified and authenticated by a botanist. Extraction method was described previously (13-15). In this method, U.dioica leaves (200 g) were dried, grinded and powdered. The powder was dissolved in 400cc Ethanol 70% (C_2_H_5_OH). The obtained solution was preserved in the hot water bath 35^o^C under dark condition. 

Then, the solution was gradually poured onto Buchner funnel filter paper and cleared by vacuum pump. It was then transferred to the rotary machine to be concentrated and to remove the extra solvent. Separation process was continued until the concentrated extract was obtained. The obtained extract weighted 8.5 g. The obtained extract was diluted by normal saline to prepare different doses. The nicotine solution (C_10_H_14_N_2_) was purchased from Merk Company (Merk-Germany). This solution was diluted by normal saline for administration.

**Animals**

This experimental study was conducted under approval of Ethics Committee of Kermanshah University of Medical Sciences. (Certificate No.91175). In this study fifty six BALB/c male mice with weight range of 25-30 g were purchased from Tehran Razi Institute. Animals were kept in the temperature of 22±2^o^C, under controlled environmental conditions, 12/12 h light/dark cycle and free access to water and food (13). 

The mice were randomly assigned to 8 groups (n=7). The control group was administered saline (1 ml/kg) and experimental groups were administered nicotine (0.5 ml/kg), U.dioica (10, 20, and 50 mg/kg) and U.dioica (10, 20, and 50 mg/kg) plus nicotine (0.5 ml/kg) interaperitoneally (IP) for 28 consequent days (14, 15).

**Analysis of testis weight and testosterone hormone**

The testes were carefully removed, washed in normal saline solution (0.9%), blotted, and weighed separately and the average weights were used. The collected blood was centrifuged at 25^o^C for 10 minutes with 4000 rpm to obtain the serum. The serum samples were then kept in deep freezer (-18^o^C). The blood testosterone level was analyzed by ELISA (Abcam 108666, USA) method (13). 

**Evaluation of** **spermatozoa** **characteristics**

Briefly, both cauda epididymis from each rat were placed in 2 ml of normal, pre-warmed, 37^o^C saline. Small cuts were made in both cauda epididymis where the spermatozoa were obtained and suspended in the medium DMEM/F12 containing FBS 5% which had been previously balanced in the incubator with the temperature of 37^o^C and CO_2_ 5% (13). The prepared suspension was used for the analysis of sperm parameters including: sperm progressive motility, count, and morphology. 

The sperm motility was assessed in 4 levels according to certain criteria: (a) quick progressive motility in direct line, (b) slow progressive motility in direct or indirect line, (c) no progressive motility and (d) no motility (16). To count the sperm cells, a small amount of the diluted suspension was transferred to both chambers of a Neubauer haemocytometer using a Pasteur pipette by touching the edge of the cover slip and allowing each chamber to be filled by capillary action. To examine the sperm cells morphology, smear was prepared from the samples and was stained and investigated by Papanicolaou method (13, 17). 

To determine the motility, one drop of the sperm cells suspension was placed on the chamber and the motile and immotile sperm cells were analyzed by microscope with magnification 40x (16, 18).

**Histological analysis**

The testes were preserved in neutral buffered formalin 10% and embedded in paraffin. Five-micron thick sections were prepared and stained with hematoxylin and eosin (HE). The specimens were examined under Olympus/3H light microscope. More than 20 sections were prepared from each block. The sections numbered 5, 10, 15, and 20 were selected and photographed separately from three random views. Seminiferous tubules diameter was measured by Motic camera and software (Moticam 2000, Spain). The mean of seminiferous tubules diameter (µm) was determined for each testis (13).

**Statistical analysis**

All the quantitative data were presented as mean±SD. One-way analysis of variance (ANOVA) followed by LSD post-hoc test were performed to determine the statistical significance between different groups using SPSS software (Statistical Package for the Social Sciences, version 16.0, SPSS Inc, Chicago, Illinois, USA). P<0.05 was considered significant.

## Results

**Weight of testis**

**Figure 1:**
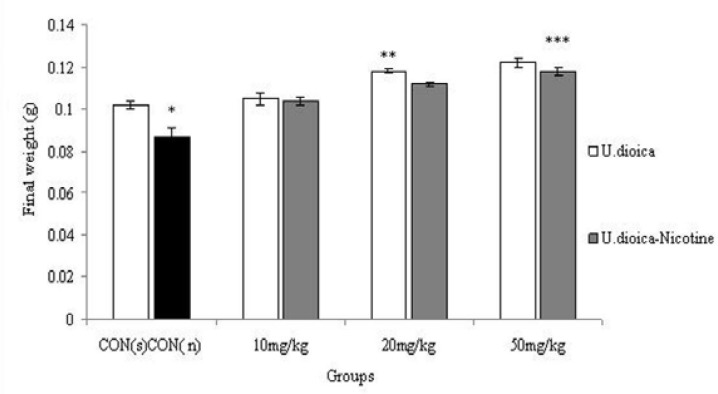
Correlation analysis between treatment groups ( Nicotine, U.dioica, and U.dioica-nicotine) in Balb/c mice and testis weight. **CON (n)**: effect of nicotine (0.5 ml/kg) on testis weight considered as control for U.dioica-nicotine group as well. **CON (s)**: control group with saline administration.* Significant decrease of testis weight in nicotine group compared to saline group (p<0.001). ** Significant increase in all groups compared to control group (p<0.001). *** Significant increase in 20 and 50 mg/kg groups compared to control group (p=0.04).

In the present study, the effective dose of nicotine (0.5 ml/kg) caused a significant decrease in the testis weight of the mice compared to control (saline) group (p<0.001). Testis weights were significantly increased in treated animals with U.dioica in 20 and 50 mg/kg doses in comparison with control (saline) group (p<0.001). Further, higher doses of U.dioica-nicotine caused a significant increase in testis weight in all treated groups in comparison with control group (nicotine) group (p=0.04) (Figure 1).

**Sperm count, motility and morphology**

The mean of sperm progressive motility, count, and normal morphology of sperm cells decreased significantly in Nicotine (0.5 ml/kg) treated group compared to control (saline) group (p<0.001). 

**Figure 2:**
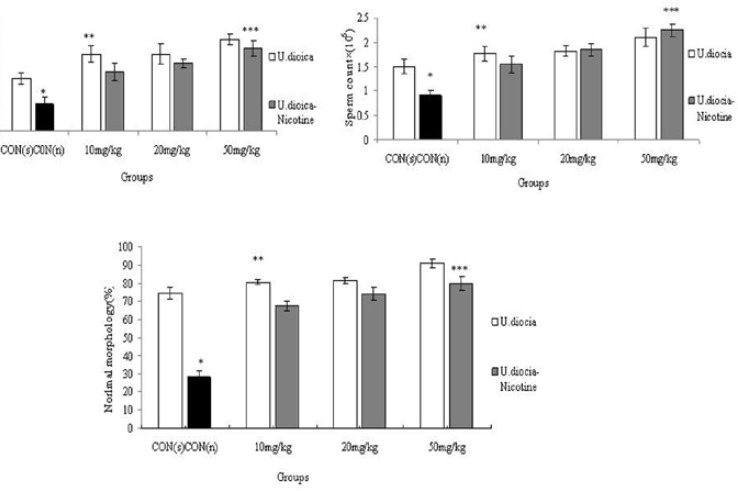
Correlation analysis between treatment groups ( Nicotine, U.dioica, and U.dioica-nicotine) in Balb/c mice and normal morphologh. **CON (n)**: effect of nicotine (0.5 ml/kg) considered as control for U.dioica-nicotine groups as well. **CON (s)**: Control group with saline administration. *Significant decrease in nicotine group compared to saline (p<0.001). ** Significant increase in all groups compared to control group (p<0.001). *** Significant increase in all groups compared to control group (p=0.04).

However, motility, count and normal morphology of sperm cells were significantly increased in all treated (U.dioica) (p<0.001) and (U.dioica-nicotine) groups in comparison with control group (Nicotine) (p=0.04) (Figure 2).

**Histological study**

**Figure 3:**
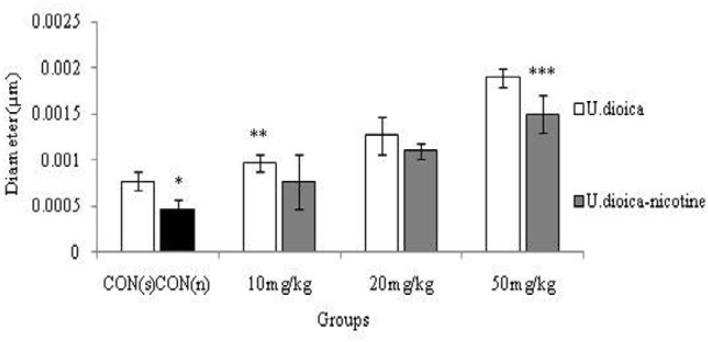
Correlation analysis between treatment groups (Nicotine, U.dioica, and U.dioica-nicotine) in Balb/c mice and seminiferous tubules diameter. **CON (n)**: Effect of nicotine (0.5 ml/kg) considered as control for U.dioica -nicotine groups as well. **CON (s)**: Control group with saline administration. *Significant decrease in nicotine group compared to saline group (p<0.001). ** Significant increase in all groups by increasing the dose (p<0.001). ***Significant increase in all groups by increasing the dose (p=0.05).

The nicotine administration (0.5 ml/kg) caused a significant decrease in the seminiferous tubules diameter in comparison with control (saline) group (p<0.001). The higher doses of U.dioica extract (p<0.001) and U.dioica-nicotine caused a significant boost in seminiferous tubules' diameter in all groups (p=0.05) (Figure 3).

**Testosterone hormone**

**Figure 4:**
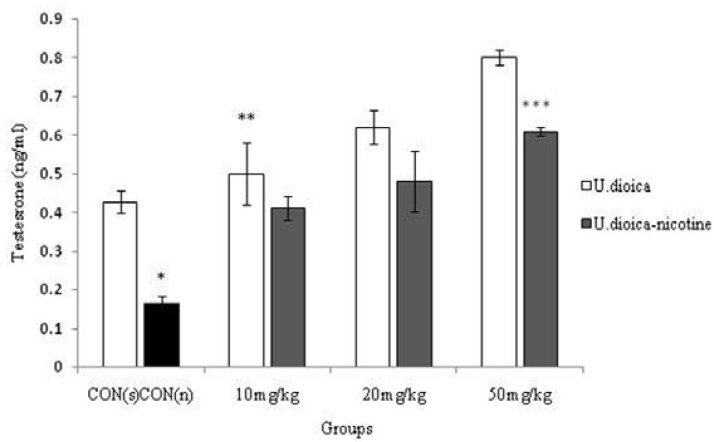
Correlation analysis between treatment groups ( Nicotine, U.dioica, and U.dioica-nicotine) in Balb/c mice and testosterone hormone level. **CON (n)**: Effect of nicotine (0.5 ml/kg) considered as control for U.dioica -nicotine groups as well. **CON (s)**: Control group with saline administration. *Significant decrease in nicotine group compared to saline group (p<0.001). ** Significant increase in all groups by increasing the dose (p<0.001). *** Significant increase in all groups by increasing the dose (p=0.04).

Nicotine (0.5 ml/kg) caused a significant increase in the testosterone hormone compared to control (saline) group (p<0.001). Increasing the dose of U.dioica extract (p<0.001) and U.dioica -nicotine revealed significant growth of testosterone hormone in all groups (p=0.04) (Figure 4).

## Discussion

The results of our experimental study revealed that administration of nicotine promoted male reproductive toxicity in mice. In the present study, the intraperitoneal injection of nicotine resulted degenerative changes in the seminiferous tubules and reduction in sperm count and motility, decrease of testosterone hormone and testis weight, and impairment of sperm cells evidence for this toxicity. This is in agreement with results of Sankako* et al* who reported residual damage on sperm concentration, motility, and morphology after cigarette smoke exposure (19). 

Neuropeptides such as neuropeptide Y and peptides hormones such as leptin could be involved in feeding-related actions of nicotine. Neuropeptide Y is a potent stimulator of feeding and could be decreased by nicotine. In contrast, leptin act as a signaling molecule and relates to the level of body fat which reserves in the hypothalamus and it could be increased by nicotine (20, 21). In addition, nicotine can also increase the release of neurotransmitters, including dopamine and serotonine, which are inhibitors food intake (22). The reductions of relative weights of testis probably occurred due to these groups' body weight increase. Also, testis weight growth might be associated with the appetite decreasing effects of nicotine that is overcome by U.dioica appetizing property (23). 

The results showed that U.dioica caused a significant change in these indices and inhibited the harmful effects induced by nicotine in reproductive parameters. These days, medicinal plants have numerous applications and one of the target tissues for plant extracts is reproductive organs such as testis and sperm parameters (13). The detrimental effects of nicotine exposure on seminiferous tubules might be due to overproduction of reactive oxygen species (ROS) that leads to excessive lipid per-oxidation. This process might eventually result in structural damage and dysfunction of the cell. As a result of excessive ROS, mutations in mitochondrial genome might occur and could disturb the formation of morphologically and functionally mature spermatozoa (24). 

However, it seems that U.dioica recover of some parameters occurred during a complete spermatic cycle and progressive morphologically normal sperm. Since U.dioica plant contains various minerals such as iron and vitamins A, which is known for regulating the differentiation of epithelial cells. It seems that the parietal cells of sperm tubules in groups receiving the extract are rapidly differentiated and released from tubules (25). Therefore, the diameter of seminiferous tubules increased after treatment with U.dioica extract.

It seems that U.dioica increases the count and motility of normal sperm cells in treated groups by enhancing the anti-oxidant defense of the body (26). In fertile individuals, sperm motility levels, especially progressive sperms, are directly related to the ability of fertilization. U.dioica can act as an anti-oxidant and improve the sperm cells quality and count by increasing the expression of anti-oxidant genes in comparison with nicotine group (27). The findings obtained in this study are in line with the results of the study conducted by Matsingou et al. in which they investigated the relative per-oxidative and anti-oxidant effects of U.dioica on the nicotine-induced toxic fatty tissue. They reported that U.dioica can decrease the toxicity induced by nicotine in the fatty tissue (28). 

High levels of unsaturated fatty acids collected in the spermatogenic cells results in several dual links creation in plasma membrane which causes a decrease in cytoplasmic antioxidants, so spermatogenic cells become sensitive to oxidative damage. Therefore, oxidation of the membrane fatty acids will result in the loss of membrane fluidity and will decrease the activity of enzymes and ion channels of sperm cells (29). With regard to the fact that nicotine is one of the producers of reactive oxygen species, it seems that clearing oxidative agents by chemicals like U.dioica can help cure and prevent the incidence of the diseases associated with sperm cells. The results of the present study confirm the findings of Inić *et al* that indicated U.dioica can be used as a potent anti-oxidant substance against oxidative stress and subsequent effects (30).

Sperm cells motility is an important factor in natural fertility and low sperms cells motility is the cause of most of infertilities (31). On the other hand, increasing sperm cells motility by U.dioica in the present study may be due to the inhibition of cannabinoids’ activity, which decreases sperm cells motility by activating CB1 receptors in mature sperm cells, by U.dioica. The activity of these factors is probably increased by nicotine (32). On the other hand, sperm motility is often used as a marker of chemical-induced testicular toxicity, therefore, our experimental results suggest a gonadotoxic potential of U.dioica (33). 

One of the reasons for this effect can be explained on the basis that, ciprofloxacin interferes with the energy production process required for sperm vitality and motility. Due to these properties, it has been proved that U.dioica is able to reduce sperm parameter damage. This would suggest that U.dioica has potential healing properties against the toxic effects of nicotine. 

## Conclusion

The present study supported the contention that U.dioica can significantly improve spermatogenesis in rats. The results also suggest the healing potential of U.dioica against toxic effects of nicotine-treated male mice. However, further studies are required for a better understanding of the interaction between U.dioica and nicotine mechanism leading to changes of spermatogenesis.

## Footenote

***This articel extracted from M.Sc. thesis. (Ali Naseri)***
